# Concise Synthesis of Broussonone A

**DOI:** 10.3390/molecules200915966

**Published:** 2015-09-02

**Authors:** Hyeju Jo, Minho Choi, Mayavan Viji, Young Hee Lee, Young-Shin Kwak, Kiho Lee, Nam Song Choi, Yeon-Ju Lee, Heesoon Lee, Jin Tae Hong, Mi Kyeong Lee, Jae-Kyung Jung

**Affiliations:** 1 College of Pharmacy and Medicinal Research Center (MRC), Chungbuk National University, Cheongju 362-763, Korea; E-Mails: hjjo317@chungbuk.ac.kr (H.J.); entia727@chungbuk.ac.kr (M.C.); cheviji@gmail.com (M.V.); yhlee91@daum.net (Y.H.L.); medchem@chungbuk.ac.kr (H.L.); jinthong@chungbuk.ac.kr (J.T.H.); mklee@chungbuk.ac.kr (M.K.L.); 2 College of Pharmacy, Korea University, Sejong 339-700, Korea; E-Mail: kiholee@korea.ac.kr; 3 College of Interdisciplinary & Creative Studies, Konyang University, Nonsan 320-711, Korea; E-Mail: nschoi@konyang.ac.kr; 4 Korea Ocean Research and Development Institute, Ansan 426-44, Korea; E-Mail: yjlee@kordi.re.kr

**Keywords:** broussonone A, cross metathesis, Grubbs catalyst, oxidative dearomatization, PIFA, total synthesis

## Abstract

A concise and expeditious approach to the total synthesis of broussonone A, a *p*-quinol natural compound, has been developed. The key features of the synthesis include the Grubbs II catalyst mediated cross metathesis of two aromatic subunits, and a chemoselective oxidative dearomatizationin the presence of two phenol moieties. Especially, optimization associated with the CM reaction of *ortho*-alkoxystyrenes was also studied, which are known to be ineffective for Ru-catalyzed metathesis reactions under conventional reaction conditions because *ortho*-alkoxy group could coordinate to the ruthenium center, resulting in the potential complication of catalyst inhibition.

## 1. Introduction

Broussonone A (**1**), a *p*-quinol natural product, was first isolated by our group from stem barks of the Korean plant *Broussonetia kanzinoki* Sieb (Moraceae) [1]. This plant is one of the most abundant trees in Korea, which has been extensively used as diuretic or tonic agents [1,2,3,4,5]. Broussonone A (**1**) exhibited inhibitory activity on pancreatic lipase with IC_50_ of 28.4 μM. Due to its structural features (e.g., *p*-quinol moiety and unrevealed stereochemistry) and interesting biological property, broussonone A lends itself as a challenging synthetic target. To date, there has been no reported total synthesis of the broussonone A.

In planning our approach, we hoped to develop a versatile and practical route that would minimize protecting group manipulations and adapt a platform that leads to a variety of analogues of **1**. Herein, we report a facile synthesis of **1** in three steps from readily available starting materials, enlisting a cross metathesis (CM) of two aromatic subunits and a chemoselective oxidative dearomatization of one of two phenol groups.

## 2. Results and Discussion

**Scheme 1:**
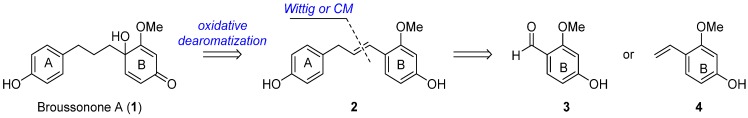
Retrosynthetic approach of broussonone A (**1**).

The crucial elements of our retrosynthetic analysis of broussonone A (**1**) are shown in Scheme 1. We envisioned that the late-stage generation of the *para*-quinol B ring could be achieved through a chemoselective oxidative dearomatization in the electron-rich subunit B of **2**. The requisite intermediate **2** could be obtained by a Wittig reaction of the aldehyde **3** or CM of the styrenyl ether **4** with a corresponding olefin, respectively.

**Scheme 2:**
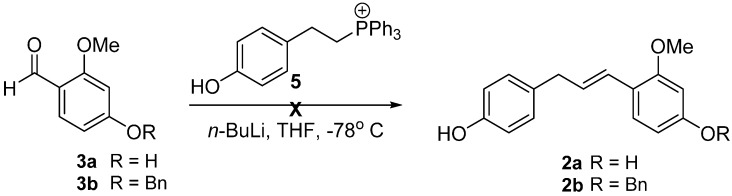
Wittig reaction to synthesize the key precursors **2**.

In order to synthesize the compound **2**, we commenced our investigation by employing a Wittig reaction between aldehydes **3** and the corresponding phosphonium salt **5**, derived from the commercially available 2-(4-hydroxyphenyl) ethanol [6,7]. However, the Wittig reaction failed to afford our desired product **2** presumably due to low reactivity of the electron-rich benzaldehydes **3** (Scheme 2) [8].

**Scheme 3:**
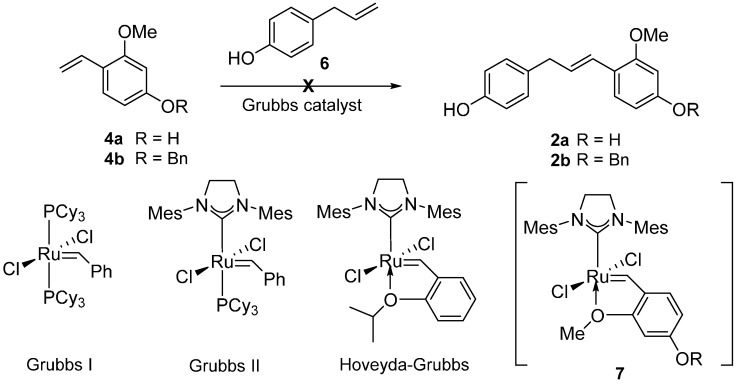
Cross metathesis (CM) reaction of 2-alkoxy styrenes **4**.

Having failed to access **2** through the Wittig reaction, we turned our attention to the CM strategy for the formation of **2**. Grubbs catalysts have been recognized as more efficient and reliable complexes widely utilized in cross metathesis reaction for a variety of alkene chemistry [9,10,11,12]. However, *ortho*-alkoxystyrenes are known to be ineffective for Ru-catalyzed metathesis reactions under conventional reaction conditions because they could readily form the highly stable Ru-chelate complex **7** discouraging the catalytic cycle [13,14]. Despite the formidable challenge ahead, we were prompted to attempt the CM reaction between **4** and **6** utilizing a Grubbs protocol since it would be the most unambiguous and rapid access to the key intermediate **2** (Scheme 3).

The allylphenol **6** was prepared following a literature procedure [15] and the 2-alkoxystyrene **4** was protected with a benzyl group. An initial CM attempt with the allylphenol **6** and 2-alkoxystyrenes **4** failed to provide the desired CM product and we observed the homodimer of **6** as the major product instead [16,17]. Thus, we were directed to use the homodimer **8** for the key CM reaction partner that was readily synthesized from **6** in 87% yield utilizing Grubbs I catalyst (2 mol %) [18,19].

Efforts were made to optimize the CM reaction outcome using **4b** and **8** as summarized in Table 1. Several ruthenium catalysts were tested and to when the Grubbs II catalyst or the Hoveyda-Grubbs II catalyst were employed, gratifyingly we obtained desired product (entries 2–4) in low yields, whereas no product formation was observed when the Grubbs I catalyst was used (entry 1). While varying the amount of catalyst loading; we obtained the best results by using 5 mol % Grubbs II catalyst (entry 4). Further increase of catalyst loading to 10 mol % produced unfavorable results such as olefin migration in **8** (entry 5). The generally preferred solvent in CM reactions such as CH_2_Cl_2_ was not suitable for our synthesis because **8** was insoluble in CH_2_Cl_2_ (entry 6). Olefin migration of **8** rather occurred in CH_2_Cl_2_ or toluene under CM condition. Among the various solvents tested, THF was the most desirable providing the desired CM product in 41% yields. Finally, controlling the stoichiometry of the substrates **8** and **4b**, we obtained the best outcome by using 2.0 equivalent of **8** and 1.0 equivalent of **4b** (entries 4, 9, and 10). We were pleased to observe that slow addition of Grubbs II catalyst over 3 h delivered **2b** in 61% isolated yield (entry 11) [18,19].

**Table 1:** Optimization of cross metathesis reaction ^a^. 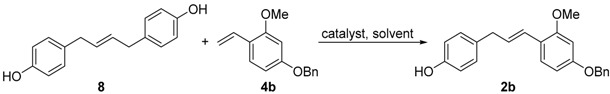

Entry	8 (equiv.)	Catalyst (mol %)	Solvent	Yield (%) ^b^
1	2.0	Grubbs I (2)	THF	NR ^c^
2	2.0	Grubbs II (2)	THF	9
3	2.0	Hoveyda-Grubbs II (2)	THF	8
4	2.0	Grubbs II (5)	THF	41
5	2.0	Grubbs II(10)	THF	37
6	2.0	Grubbs II (5)	CH_2_Cl_2_	4
7	2.0	Grubbs II (5)	DCE	12
8	2.0	Grubbs II (5)	toluene	11
9	1.0	Grubbs II (5)	THF	30
10	4.0	Grubbs II (5)	THF	36
**11**	**2.0**	**Grubbs II (5)**	**THF**	**61 ^d^**

^a^ Unless otherwise specified, all the reaction were conducted in the presence of **8**, 1.0 equiv. of **4b**, and catalyst at 70 °C. ^b^ isolated yield. ^c^ No reaction. ^d^ Catalyst was slowly added over 3 h.

**Scheme 4:**
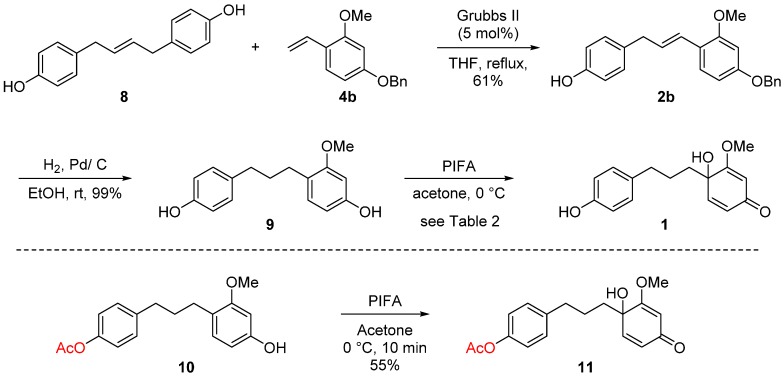
Completion of total synthesis of **1**.

Hydrogenation of **2b** with Pd/C catalyst furnished **9** [20] in 99% yield to face the final step in the total synthesis of broussonone A (Table 2). We initially anticipated the disparity in electron density of the two aromatic rings would serve as the key controlling factor for the final chemoselective dearomatization step. In recent years, hypervalent iodine reagents such as PIDA, HDIP, and PIFA have been used as oxidative reagents significantly due to their advantages including ready availability, low toxicity, ease of handling, similar activity to heavy metal catalysts, *etc.* [21,22,23,24]. The phenyliodine (III) diacetate (PIDA)-mediated oxidative dearomatization afforded broussonone A in low yields and mostly many unidentifiable by-products (entry 1). To our delight, replacement of PIDA with phenyliodine (III) bis(trifluoroacetate) (PIFA) accelerated the reaction and the oxidative conversion of B ring reached over 69% yield (38% yield of the desired compound **1**), while at the same time the reaction accompanied the second dearmoatization to yield **12** as a major contaminant in over 31% yield (entry 3). Subsequently, the effects of various solvents were also examined. In CH_3_CN and THF, **12** was identified as the sole major product (entries 4–5). Using CH_3_CN/H_2_O (2/1) or acetone as solvents did not improve the ratio of **1** and **12** (entries 1–3). When the reaction was performed in the presence of 1.0 equiv. of PIFA and 1.0 equiv. of **2** in acetone at 0 °C, the best result was obtained to provide broussonone A (**1**) in 40% isolated yield along with 31% yield of **12** (entry 6, 71% combined yield) [25]. It should be noted that the oxidative dearomatization of the protected substrates **10** provided the Ac-protected broussonone A **11** in only 55% yield. Considering the additional steps for protection and deprotection, our strategy employing the chemoselective oxidative dearomatization in final stage could be considered competitive (Scheme 4). The spectroscopic properties (^1^H- and ^13^C-NMR, HRMS) of the synthetic broussonone A were compatible with those of the natural **1** [1].

**Table 2:** Optimization of oxidative dearomatization ^a^. 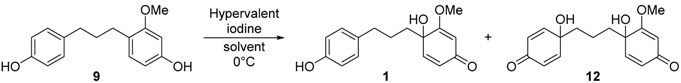

Entry	Hypervalent Iodine	Solvent	Yield (%) ^b^	
			1	12
1	PhI(OAc)_2_	CH_3_CN/H_2_O (2/1)	7	3
2	PhI(OAc)_2_	Acetone	16	27
3	PhI(OCOCF_3_)_2_	CH_3_CN/H_2_O (2/1)	38	31
4	PhI(OCOCF_3_)_2_	CH_3_CN	-	>49
5	PhI(OCOCF_3_)_2_	THF	-	>49
**6**	**PhI(OCOCF_3_)_2_**	**Acetone**	**40**	**31**
7	PhI(OCOCF_3_)_2_	Acetone/H_2_O (2/1)	22	21
8	PhI(OCOCF_3_)_2_	CH_3_OH/H_2_O (2/1)	-	31

^a^ 1.0 equiv. of **9** and 1.0 equiv. of oxidant was used and the reaction was stirred at 0 °C for 30 min. ^b^ isolated yield.

## 3. Experimental Section

### 3.1. General Information

All manipulations of compounds were performed under a nitrogen atmosphere. THF were obtained from Sigma Aldrich Co. (St. Louis, MO, USA), purified by dried over fresh Na chips. All reactant or reagent was purchased from Sigma Aldrich Co. or Tokyo Chemical Industry (Tokyo Chemical Industry, Tokyo, Japan), and used without purification. Silica gel column chromatography was performed with Silica Gel of Kieselgel 60 F_254_ plate (Merck Millipore Corporation, Darmstadt, Germany). All ^1^H-NMR and ^13^C-NMR spectra of organic products were recorded on Bruker DPX 400 MHz Spectrometer (Bruker Corporation, Karlsruhe, Germany) and Bruker AVANCE 500 MHz Spectrometer (Bruker Corporation, Karlsruhe, Germany). Data are reported as follows: chemical shift in ppm (δ), multiplicity (s = singlet, d = doublet, t = triplet, m = multiplet), coupling constant (Hz), and integration. 

### 3.2. Synthesis

*4-Allyl phenol* (**6**). 4-Allylanisole (0.768 mL, 5.00 mmol) was dissolved in CH_2_Cl_2_ and then BBr_3_ (5.50 mL, 5.50 mmol) was added at 0 °C. Then the mixture was stirred at same temperature for 1 h. After the completion of the reaction, the reaction mixture was quenched with H_2_O and extracted with CH_2_Cl_2_. The combined organic phase were dried with Na_2_SO_4_ and concentrated *in vacuo* to give a crude product and it was purified by column chromatography on silica gel (EtOAc/hexanes = 1/3) to afford 4-allylphenol **6** (654 mg, 97.5%). ^1^H-NMR (CDCl_3_, 400 MHz) δ 7.06 (d, 2H, *J* = 8.2 Hz), 6.78 (d, 2H, *J* = 8.4 Hz), 5.95 (m, 1H), 5.07 (m, 2H), 3.33 (d, 2H, *J* = 6.7 Hz).

*(E)-4,4'-(But-2-ene-1,4-diyl)diphenol* (**8**). To a stirred solution of 4-allylphenol (**6**) (327 mg, 2.44 mmol) in dried CH_2_Cl_2_ was added Grubbs I catalyst (40.1 mg, 48.8 mmol) and the mixture was heated at reflux for 4.5 h. The solvent was removed under reduced pressure and purified by column chromatography on silica gel (EtOAc/hexanes = 1/4) to afforddimer **8** (256 mg, 87.3%). ^1^H-NMR (CD_3_OD, 400 MHz) δ 6.95 (d, 4H, *J* = 8.5 Hz), 6.68 (d, 2H, *J* = 8.5 Hz), 5.55 (m, 2H), 3.22 (d, 4H, *J* = 5.0 Hz);^13^C-NMR (CD_3_OD, 125 MHz) δ 155.1, 131.6, 130.3, 129.0, 114.7, 37.6; MS *m*/*z* (M + H)^+^ calculated for C_16_H_16_O_2_: 240.1; found 240.1.

*4-(Benzyloxy)-2-methoxybenzaldehyde* (**3a**). To a solution of 4-hydroxy-2-methoxybenzaldehyde (**3**) (200 mg, 1.31 mmol) in DMF was added K_2_CO_3_ (543 mg, 3.93 mmol) under N_2_ atmosphere and mixture was stirred at room temperature for 30 min. BnBr (0.312 mL, 2.62 mmol) was added to reaction mixture and stirred for 1.5 h. After the completion of the reaction, the reaction mixture was diluted with EtOAc and washed with H_2_O. The organic phase were dried with Na_2_SO_4_ and concentrated *in vacuo* purified by column chromatography on silica gel using (EtOAc/hexanes = 1/2) to afford aldehyde **3a** (304 mg, 95.7%). ^1^H-NMR (CDCl_3_, 400 MHz) δ 7.81 (d, 1H, *J* = 8.6 Hz), 7.36 (m, 5H), 6.62 (dd, 1H, *J* = 8.7, 2.2 Hz), 6.54 (d, 1H, *J* = 2.2 Hz), 5.14 (s, 2H), 3.89 (s, 3H).

*4-(Benzyloxy)-2-methoxy-1-vinylbenzene* (**4b**). Under N_2_ atmosphere, a solution of *t*-BuOK (1.0 M solution in THF, 6.00 mL, 6.00 mmol) was added methyltriphenylphosphonium bromide (1.29 g, 3.60 mmol) at 0 °C and stirred at room temperature for 30 min. Then, the reaction mixture was cooled to 0 °C and a solution of **4a** (291 mg, 1.20 mmol) in dry THF was added to reaction mixture and stirred for 4 h. After the completion of the reaction, the reaction mixture was quenched with saturated NH_4_Cl and extracted with CH_2_Cl_2_. The organic phase were dried with Na_2_SO_4_ and concentrated *in vacuo* and purified by column chromatography on silica gel (EtOAc/hexanes = 1/3) to afford styrene **4b** (176 mg, 61.2%).^1^H-NMR (CD_3_OD, 400 MHz) δ 7.35 (m, 6H), 6.90 (dd, 1H, *J* = 17.8 Hz), 6.53 (m, 2H), 5.57 (dd, 1H, *J* = 17.8, 1.7 Hz), 5.04 (dd, 1H, *J* = 11.2, 1.7 Hz), 5.04 (s, 2H), 3.76 (s, 3H); ^13^C-NMR (CD_3_OD, 125 MHz) δδ 159.9, 157.9, 137.3, 131.1, 128.1, 127.5, 127.2, 126.7, 119.8, 110.7, 105.9, 98.7, 69.7, 54.6; MS *m*/*z* (M + H)^+^ calculated for C_16_H_16_O_2_: 240.1; found 240.1.

*(E)-4-(3-(4-(Benzyloxy)-2-methoxyphenyl)allyl)phenol* (**2b**). To a stirred solution of (*E*)-4,4'-(but-2-ene-1,4-diyl)diphenol (**8**) (200 mg, 0.832 mmol) and 4-(benzyloxy)-2-methoxy-1-vinylbenzene (**4b**) (100 mg, 0.416 mmol) in anhydrous THF was added Grubbs II catalyst (17.7 mg, 0.416 mmol) and the mixture was heated at reflux for 16 h. After the completion of the reaction, the solvent was removed under reduced pressure and the resulting residue was subjected to silica gel chromatography (EtOAc/hexanes = 1/6) to afford alkene **2b** (87.8 mg, 61.0%).^1^H-NMR (CD_3_OD, 400 MHz) δ 7.43–7.27 (m, 6H), 7.01 (d, 2H, *J* = 8.6 Hz), 6.69 (d, 2H, *J* = 8.6 Hz), 6.55 (m, 3H), 6.16 (m, 1H), 5.05 (s, 2H), 3.78 (s, 3H), 3.38 (d, 2H, *J* = 6.0 Hz); ^13^C-NMR (CDCl_3_, 100 MHz) δ 159.2, 157.5, 153.8, 136.9, 133.0, 129.7, 128.6, 128.2, 128.0, 127.5, 127.2, 125.1, 119.9, 115.2, 105.7, 99.4, 70.2, 55.5, 39.0; MS *m*/*z* (M + H)^+^ calculated for C_23_H_22_O_3_: 346.2; found 346.2.

*4-(3-(1-Hydroxy-2-methoxy-4-oxocyclohexa-2,5-dien-1-yl)propyl)phenyl acetate* (**11**). To a solution of 4-(3-(4-hydroxy-2-methoxyphenyl)propyl)phenyl acetate (**10**) (30 mg, 0.10 mmol) in acetone (0.5 mL) at 0 °C was treated with PIFA (43 mg, 0.1 mmol) and stirred for 10 min at 0 °C. After the completion of the reaction, the reaction mixture was diluted with CH_2_Cl_2_ and washed with saturated NaHCO_3_ solution. The aqueous phase was extracted with CH_2_Cl_2_. The combined organic extracts were dried with Na_2_SO_4_, concentrated *in vacuo* and purified by column chromatography on silica gel (EtOAc/hexanes = 1/1) to afford *p*-quinol **11** (17 mg, 55%). ^1^H-NMR (CD_3_OD, 400 MHz) δ 7.11 (d, 2H, *J* = 8.6 Hz), 6.98 (d, 2H, *J* = 8.5 Hz), 6.57 (d, 1H, *J* = 10.0 Hz), 6.16 (dd, 1H, *J* = 10.0, 1.6 Hz), 5.51 (d, 1H, *J* = 1.6 Hz), 3.75 (s, 3H), 2.55 (m, 2H), 2.28 (s, 3H), 1.95 (m, 1H), 1.80 (m, 1H), 1.37 (m, 1H), 0.84 (m, 1H); HRMS *m*/*z* (M + H)^+^ calculated for C_18_H_20_O_5_: 317.1384; found 317.1386.

*4-(3-(4-Hydroxyphenyl)propyl)-3-methoxyphenol* (**9**). To a solution of alkene **2b** (101 mg, 0.292 mmol) and 10% Pd/C (12.4 mg, 0.117 mmol) in EtOH, hydrogen gas was fluxed for 10 min at room temperature. The reaction mixture was stirred under H_2_ atmosphere for 16 h at room temperature, then filtered over Celite, the solvent was discarded and the residue purified by column chromatography on silica gel (EtOAc/hexanes = 1/6), to afford phenol **9** (74.7 mg, 99.0%). ^1^H-NMR (CD_3_OD, 400 MHz) δ 6.95 (d, 2H, *J* = 8.5 Hz), 6.84 (d, 1H, *J* = 8.1 Hz), 6.66 (d, 2H, *J* = 8.5 Hz), 6.36 (d, 1H, *J* = 2.3 Hz), 6.27 (dd, 1H, *J* = 8.1, 2.3 Hz), 3.73 (s, 3H), 2.47 (m, 4H), 1.75 (m, 2H); ^13^C-NMR (CD_3_OD, 125 MHz) δ 158.3, 156.3, 154.8, 133.4, 129.7, 128.8, 121.4, 114.6, 106.1, 98.4, 54.2, 34.4, 32.2, 28.9; HRMS *m*/*z* (M + H)^+^ calculated for C_16_H_18_O_3_: 259.1334; found 259.1316.

*Broussonone A* (**1**)*.* To a solution of 4-(3-(4-hydroxyphenyl)propyl)-3-methoxyphenol (**9**) (26.0 mg, 0.10 mmol) in acetone (1.0 mL) at 0 °C was treated with PIFA (42.0 mg, 0.10 mmol) and stirred for 30 min at 0 °C. The reaction mixture was diluted with CH_2_Cl_2_ and washed with saturated NaHCO_3_ solution. The aqueous phase was extracted with CH_2_Cl_2_. The combined organic extracts were dried with Na_2_SO_4_, concentrated *in vacuo* and purified by column chromatography on silica gel (5% MeOH in CH_2_Cl_2_) to afford the synthetic broussonone A (**1**) (11.0 mg, 40%). ^1^H-NMR (CD_3_OD, 500 MHz) δ 6.91 (d, 2H, *J* = 8.5 Hz), 6.66 (d, 2H, *J* = 8.5 Hz), 6.61 (d, 1H, *J* = 10.0 Hz), 6.07 (dd, 1H, *J* = 10.0 Hz, 1.7 Hz), 5.52 (d, 1H, *J* = 1.7 Hz), 3.76 (s, 3H), 2.46 (m, 2H), 1.95 (m, 1H), 1.70 (m, 1H), 1.49 (m, 2H); ^13^C-NMR (CD_3_OD, 125 MHz) δδ 188.8, 177.1, 155.1, 148.7, 132.4, 128.9, 126.7, 114.7, 100.7, 70.8, 55.2, 37.6, 34.2, 25.3; HRMS *m*/*z* (M + H)^+^ calculated for C_16_H_18_O_4_: 274.1267; found 274.1267.

For more details of NMR spectra, please see Supplementary Materials Figures S1–S17. 

## 4. Conclusions

In summary, we have successfully demonstrated the first total synthesis of broussonone A in three steps from the known compound **4b** with a 24% overall yield. The key features of this synthetic route involve the following: (1) the Grubbs II catalyst mediated cross metathesis of two aromatic subunits; (2) the chemoselective oxidative dearomatization in the presence of two phenol moieties. These studies provide a timely contribution to the development of a practical synthetic approach to a variety of broussonone A analogues. With this practical synthesis of broussonone A now in hand, the intensive exploration of the absolute configuration of the broussonone A as well as its structural analogues will be extended.

## Supplementary Materials

Supplementary materials can be accessed at: http://www.mdpi.com/1420-3049/20/09/15966/s1.
