# Supplementary material for: Concise Synthesis of Broussonone A
Source: Molecules. 2015 Sep 2;20(9):15966–75. doi: 10.3390/molecules200915966 (PMC6331890; doi:10.3390/molecules200915966)
Supplement: Supplementary file 1 [file molecules-20-15966-s001.pdf]

Supplementary Materials

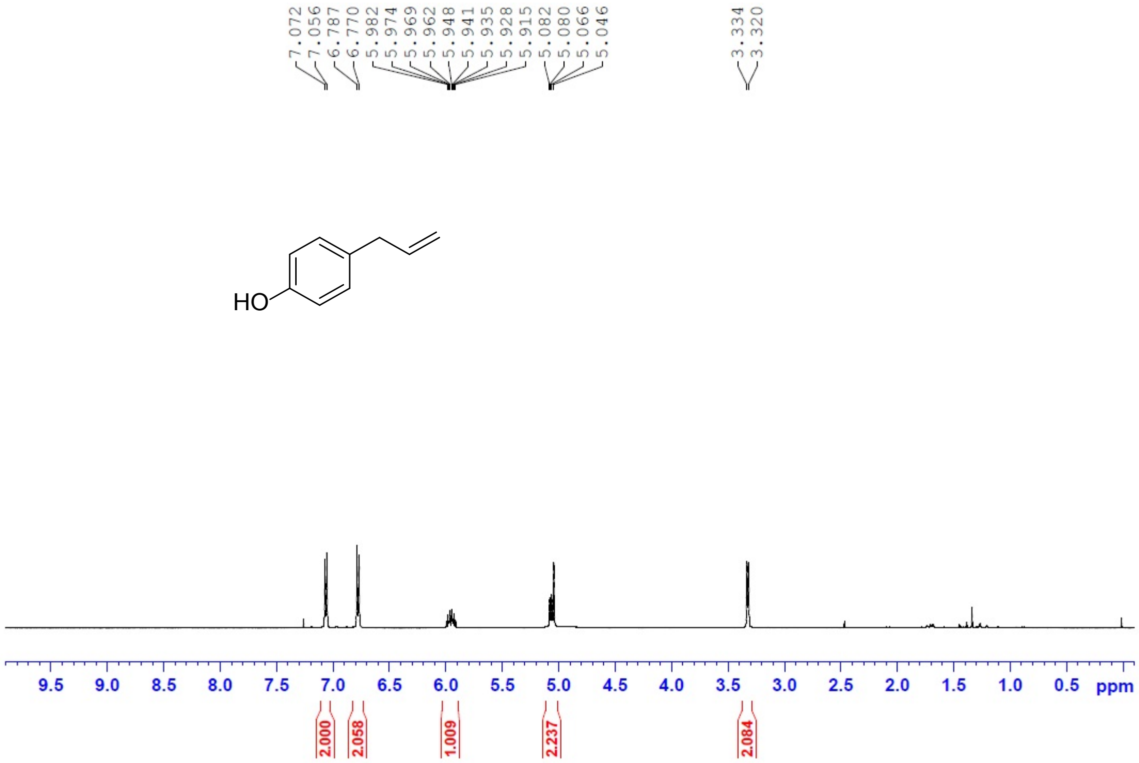

Figure S1. ^{1}H-NMR spectra of **6**.

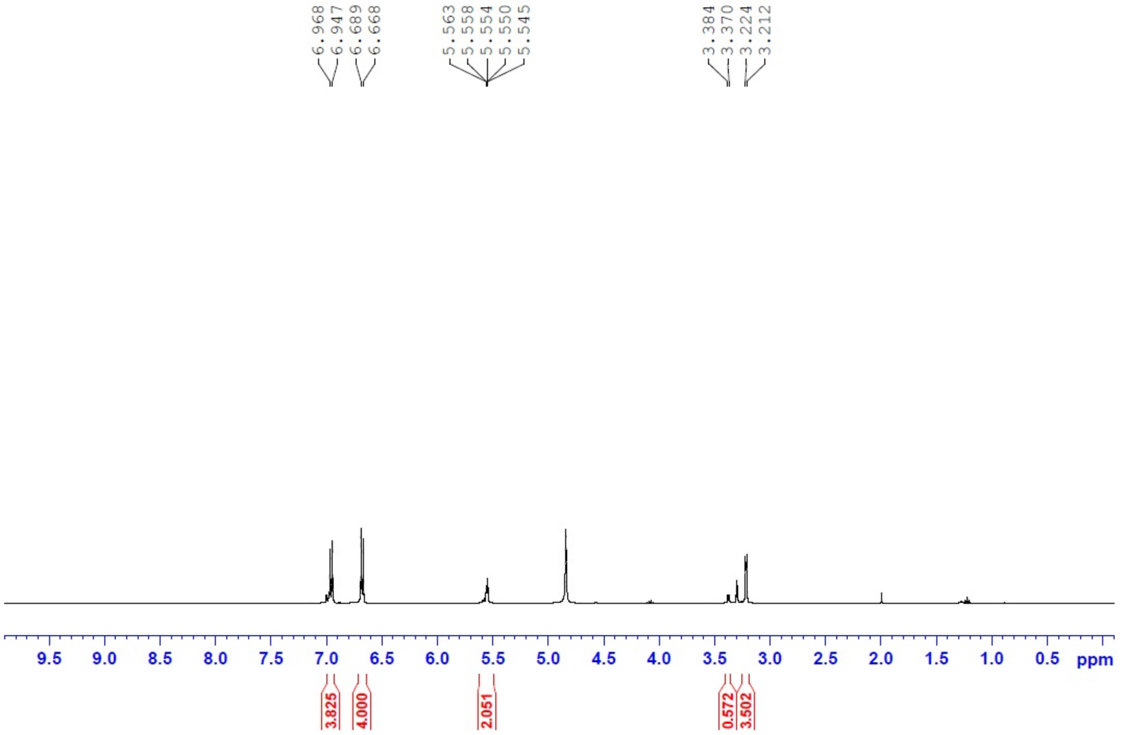

Figure S2. ^{1}H-NMR spectra of **8**.

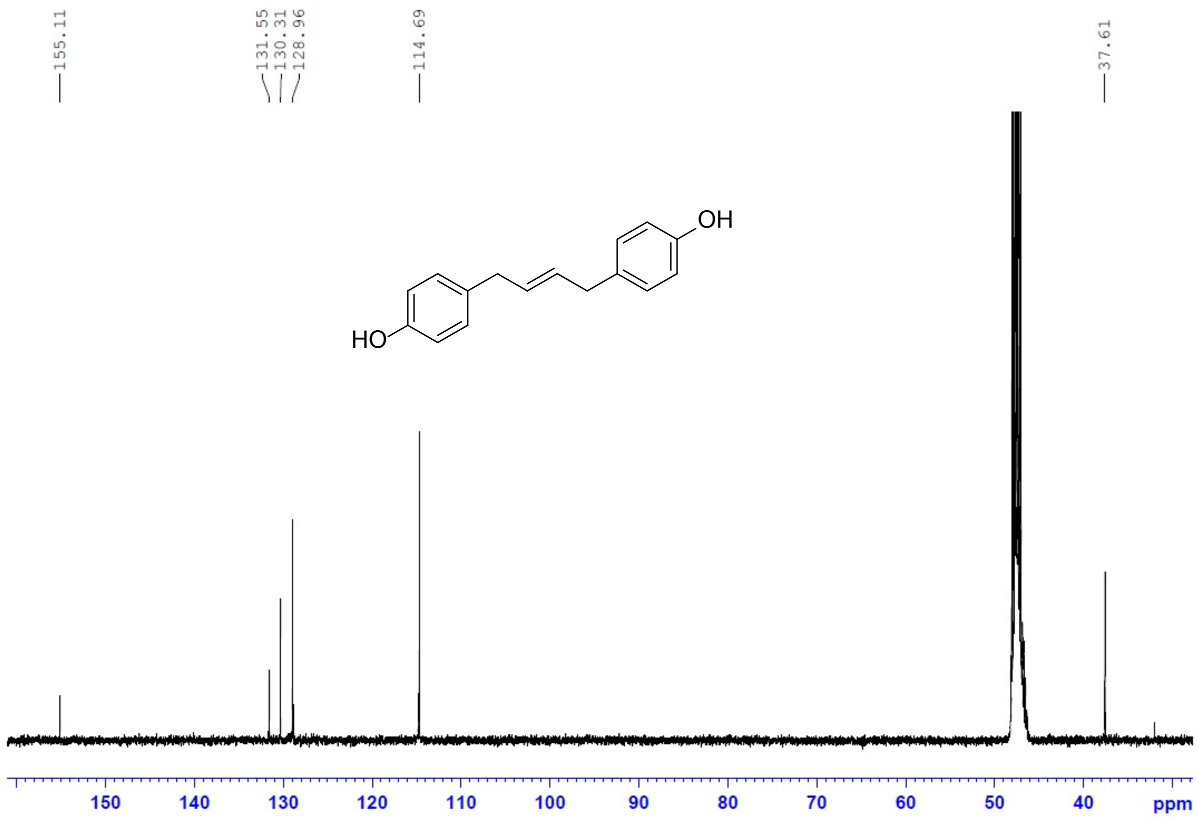

Figure S3. ^{13}C-NMR spectra of **8**.

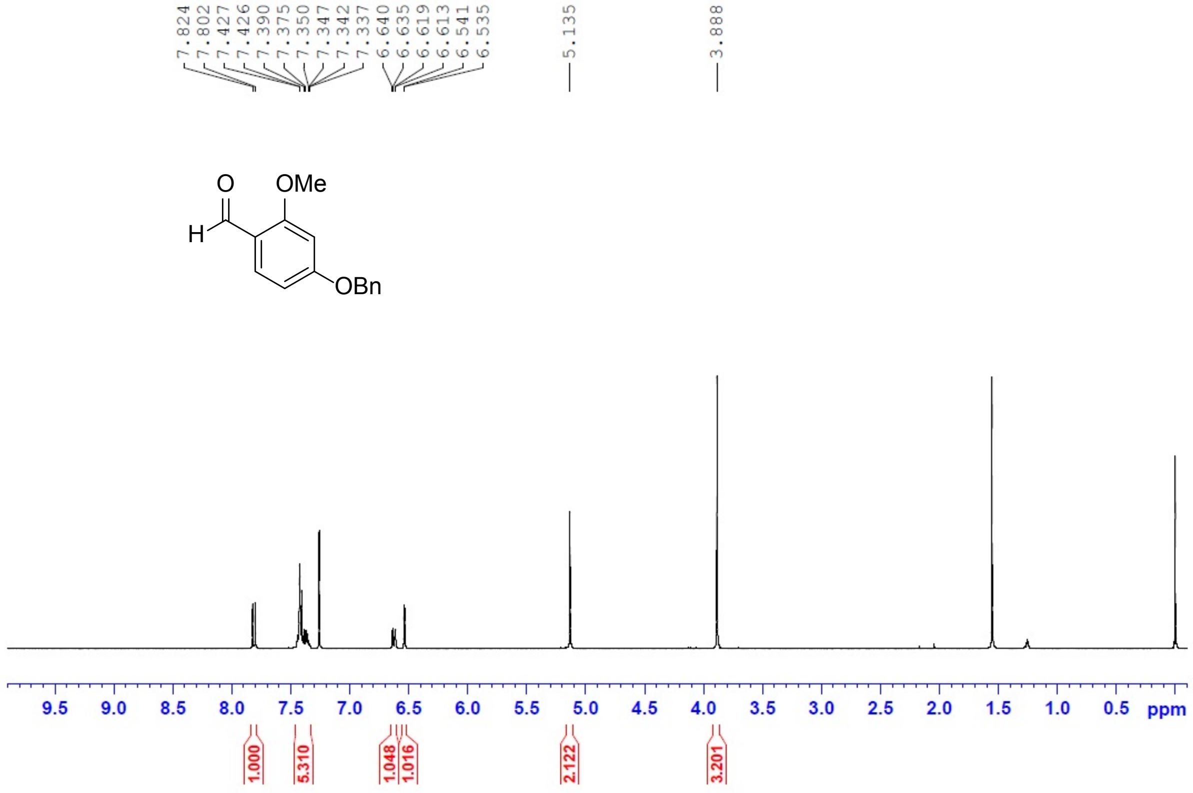

Figure S4. ^{1}H-NMR Spectra of **3a**.

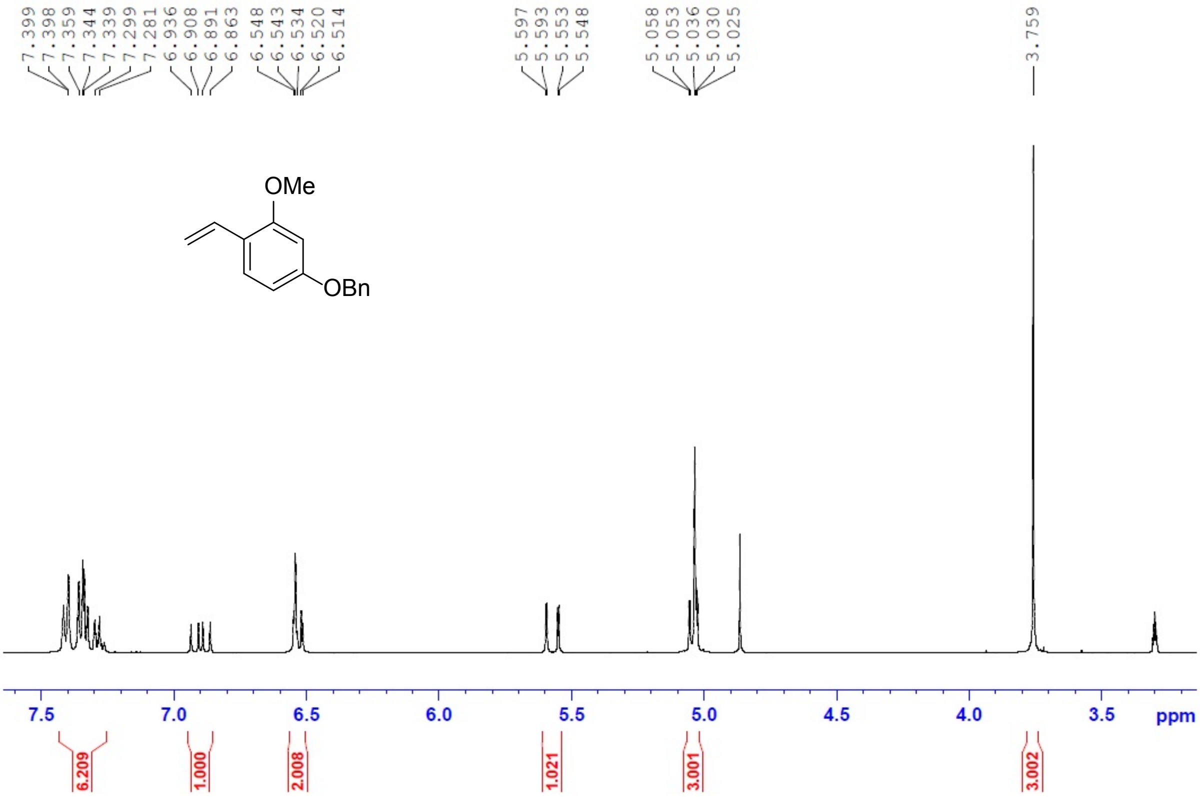

Figure S5. ^{1}H-NMR spectra of **4b**.

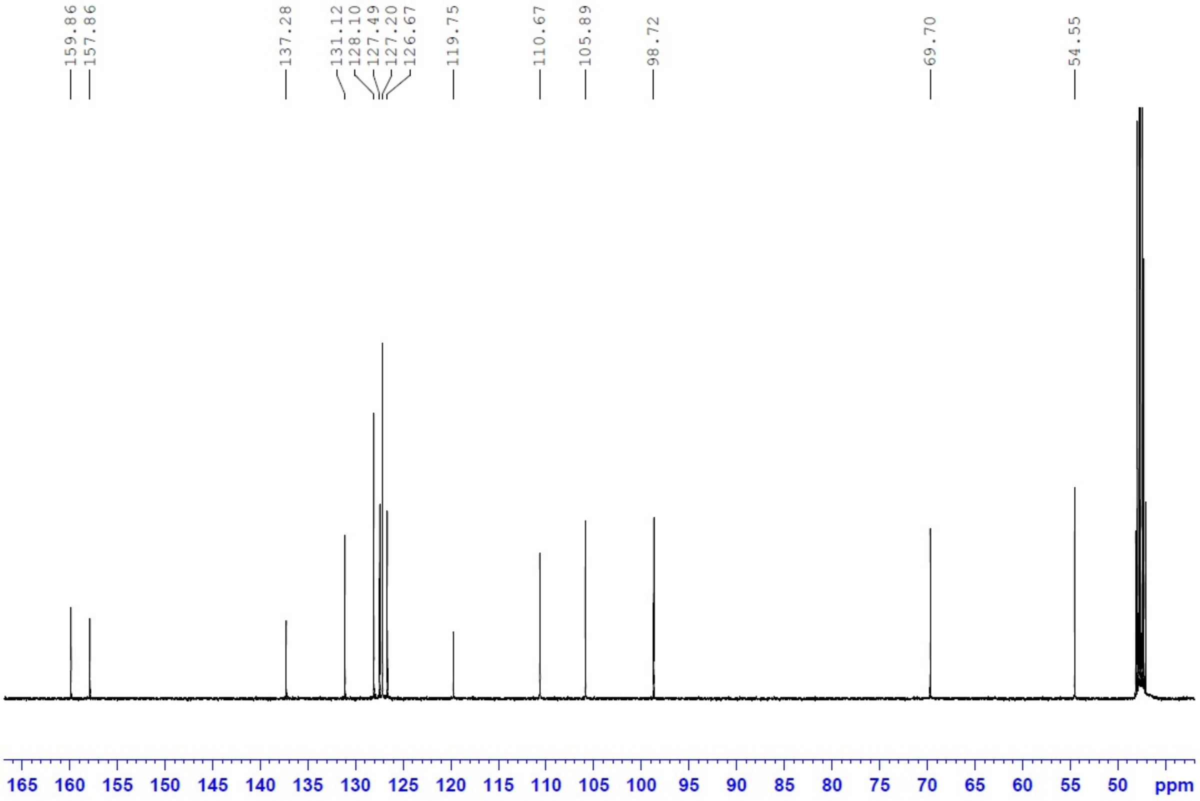

Figure S6. ^{13}C-NMR spectra of **4b**.

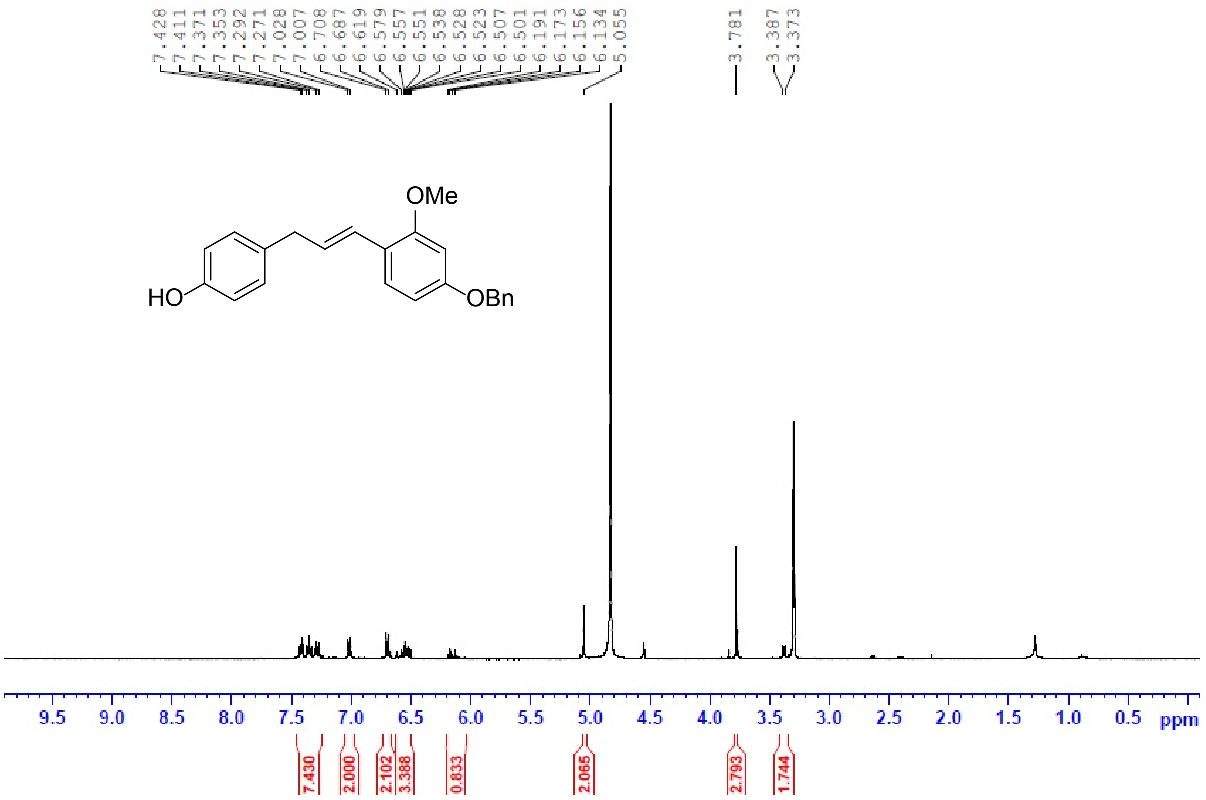

Figure S7. ^{1}H-NMR spectra of **2b**.

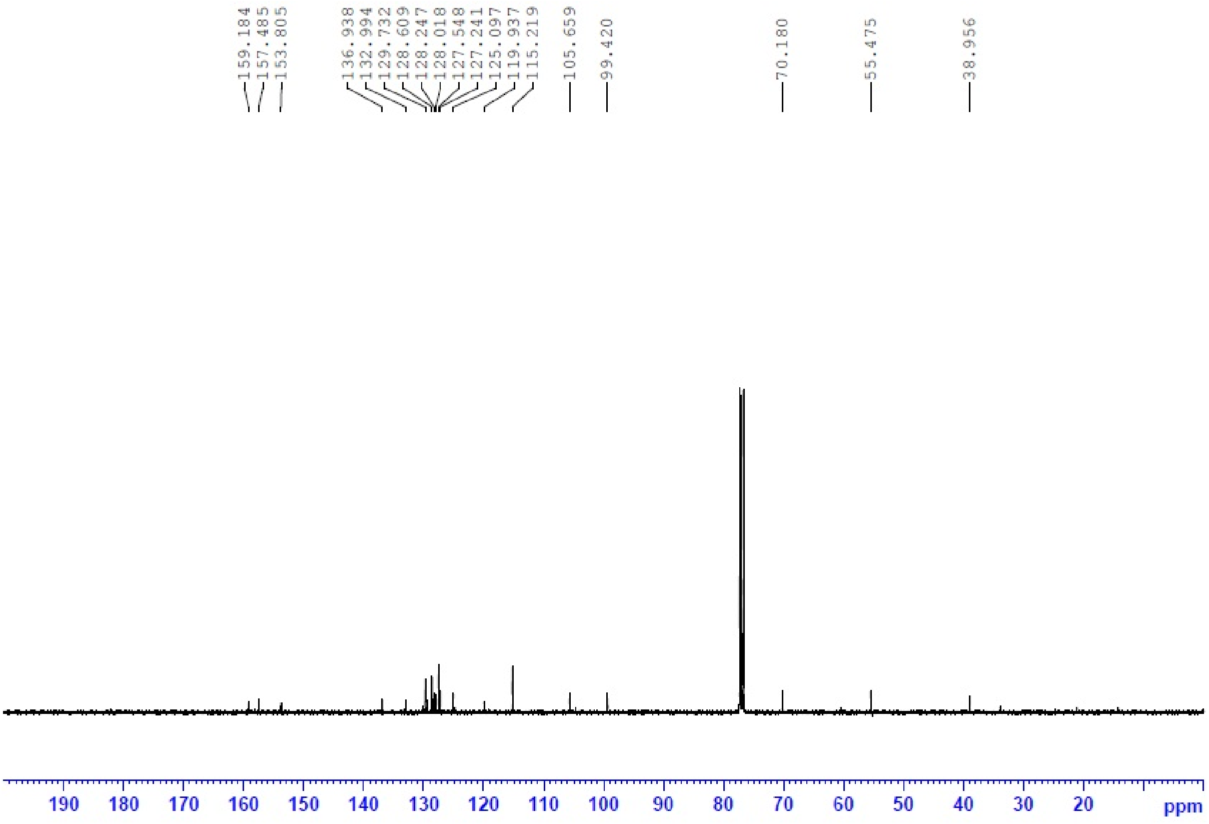

Figure S8. ^{13}C-NMR spectra of **2b**.

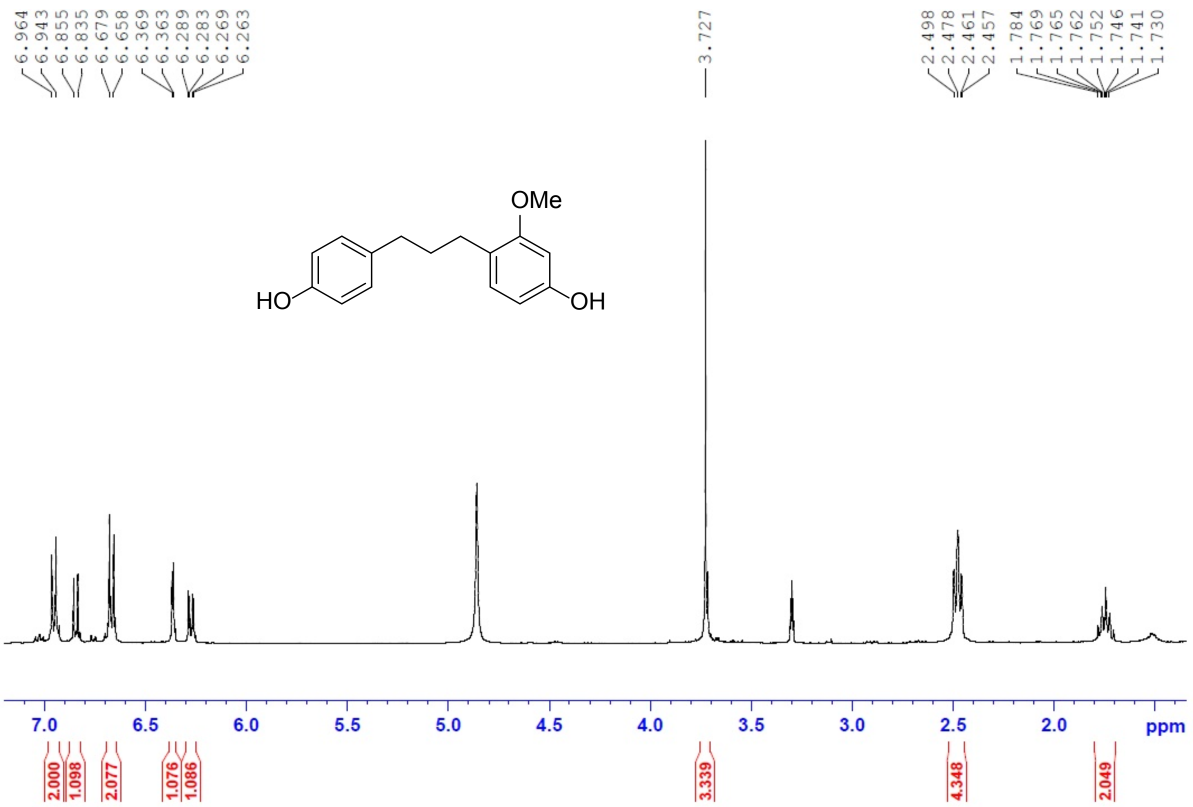

Figure S9. ^{11}H-NMR spectra of **9**.

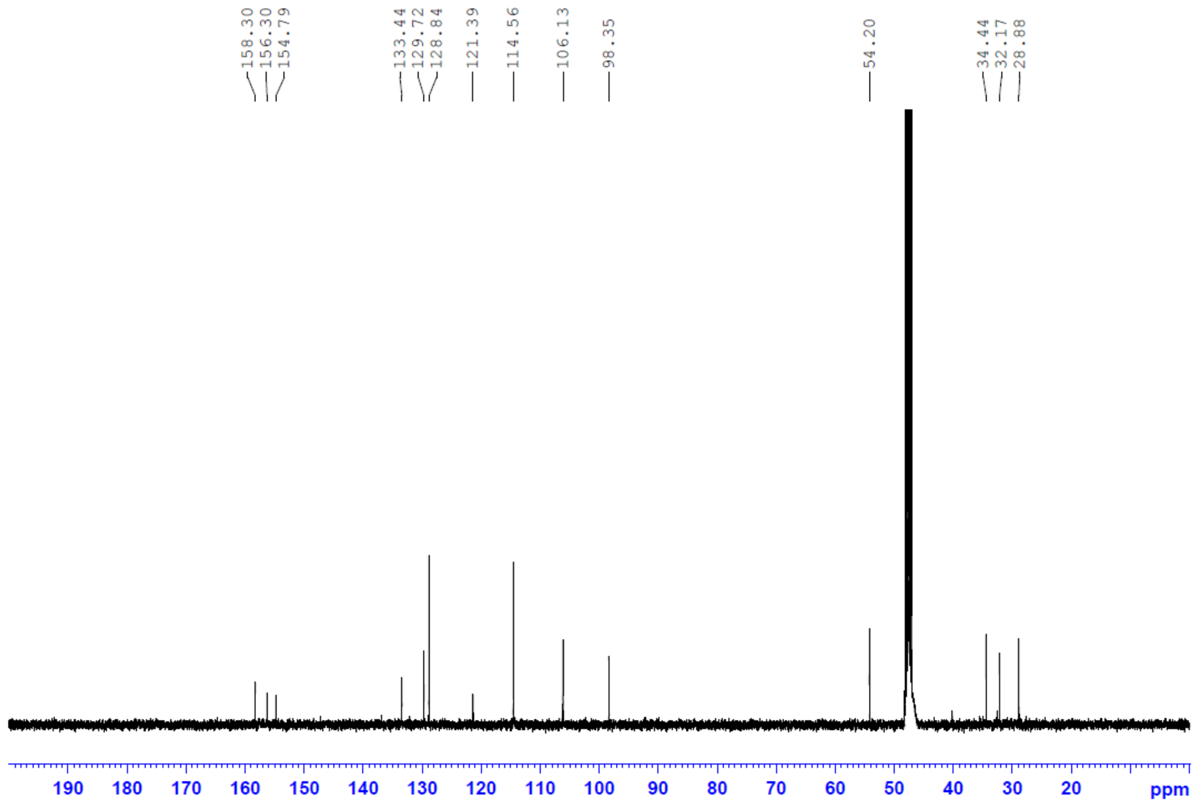

Figure S10. ^{1}C-NMR spectra of **9**.

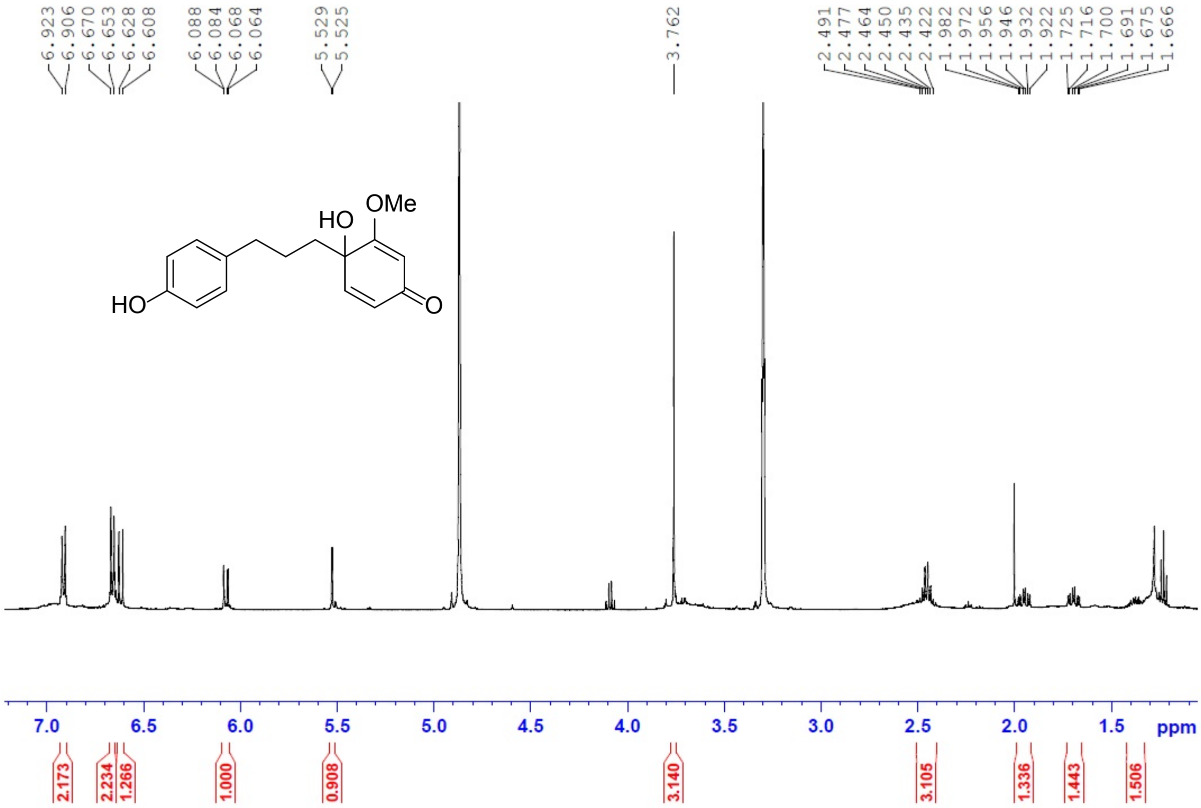

Figure S11. ^{1}H-NMR spectra of synthetic broussone A.

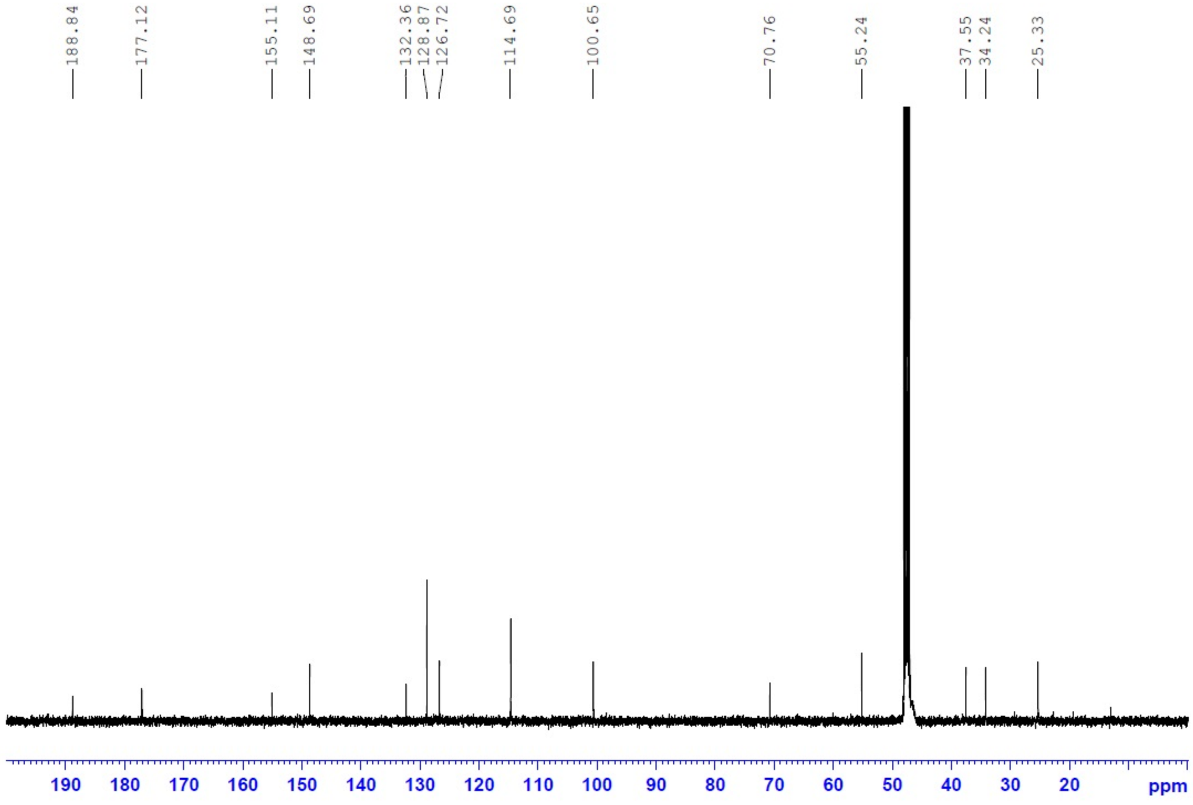

Figure S12. ^{13}C-NMR spectra of synthetic broussone A.

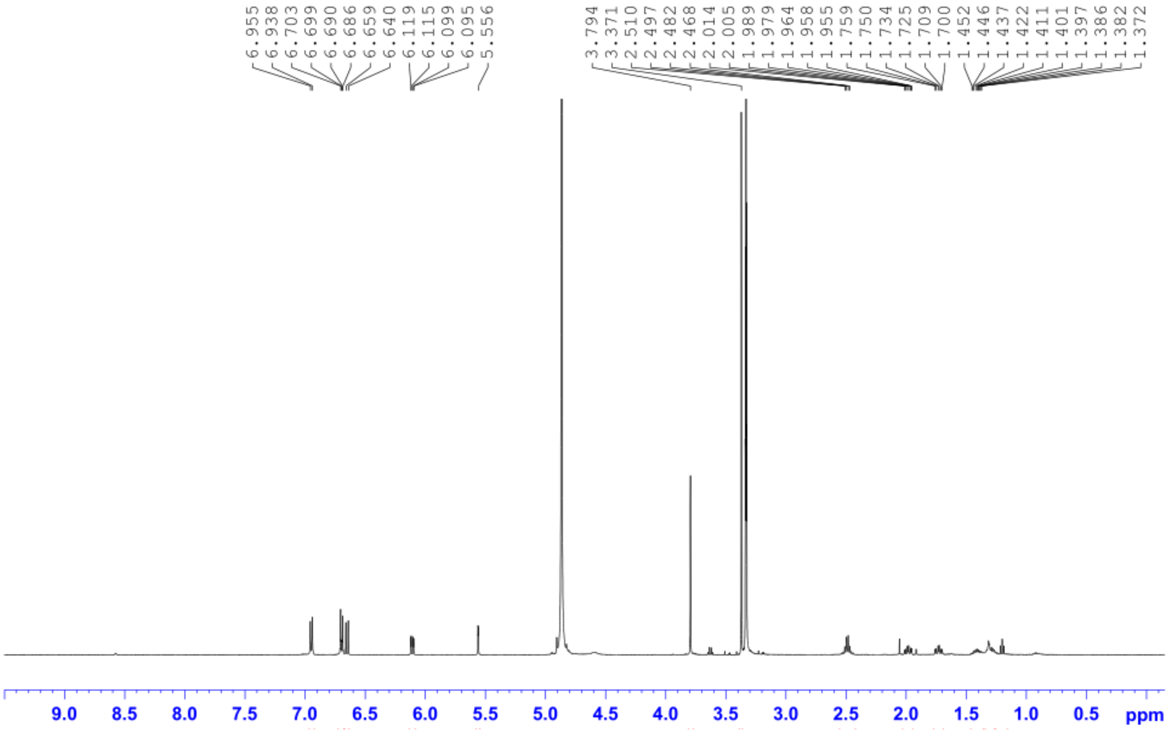

Figure S13. ^{1}H-NMR spectra of natural broussonone A.

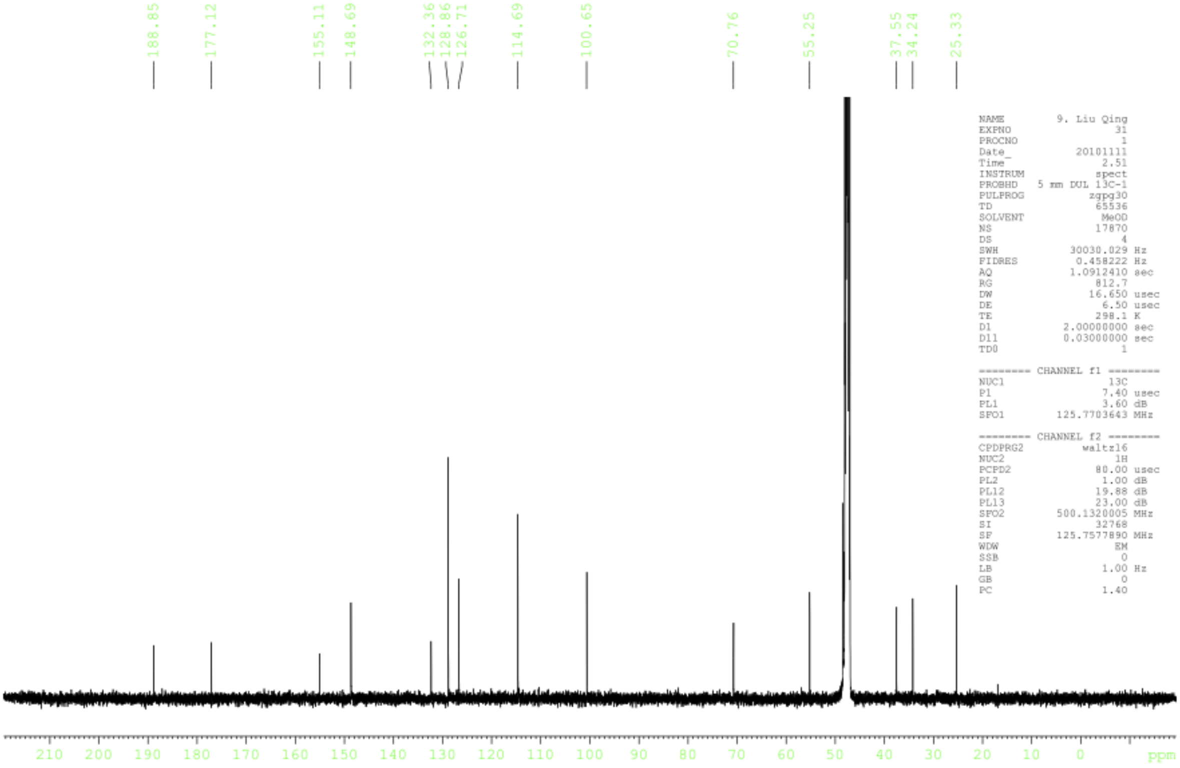

Figure S14. ^{13}C-NMR spectra of natural broussonone A.

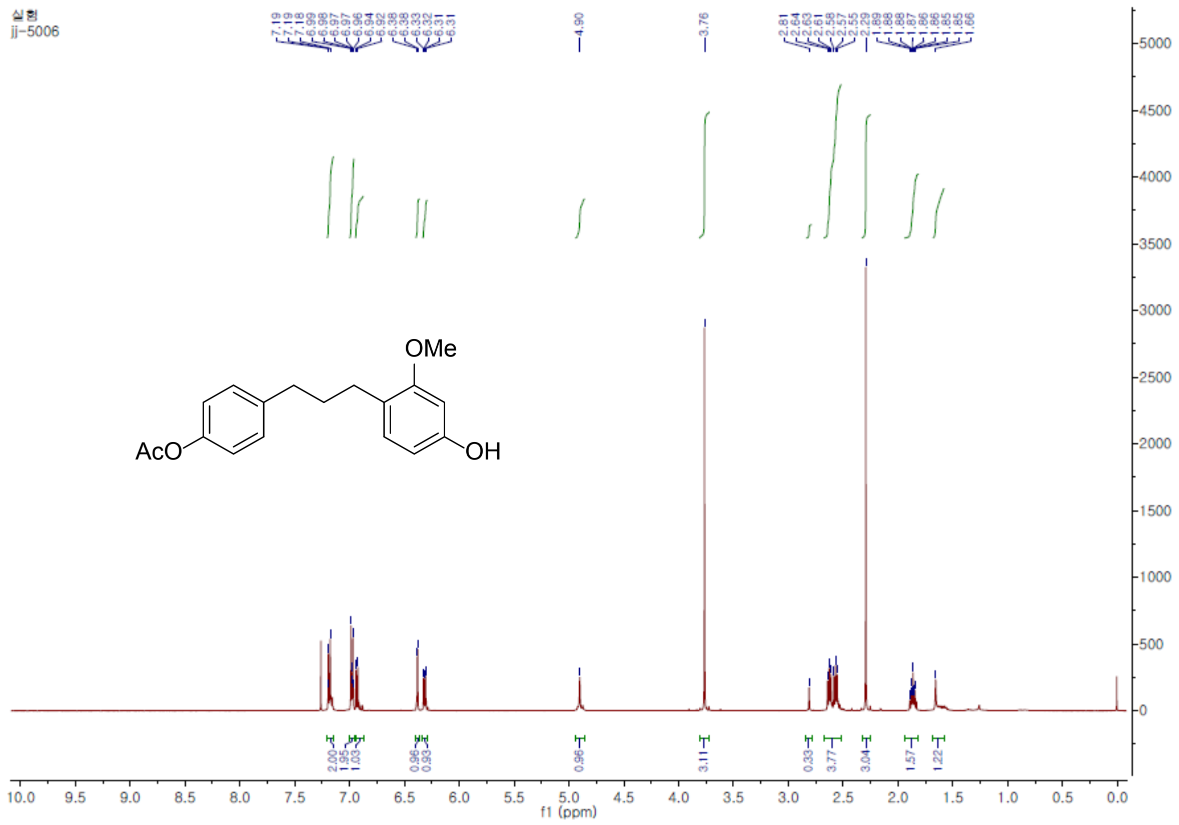

Figure S15. ^{1}H-NMR spectra of **10**.

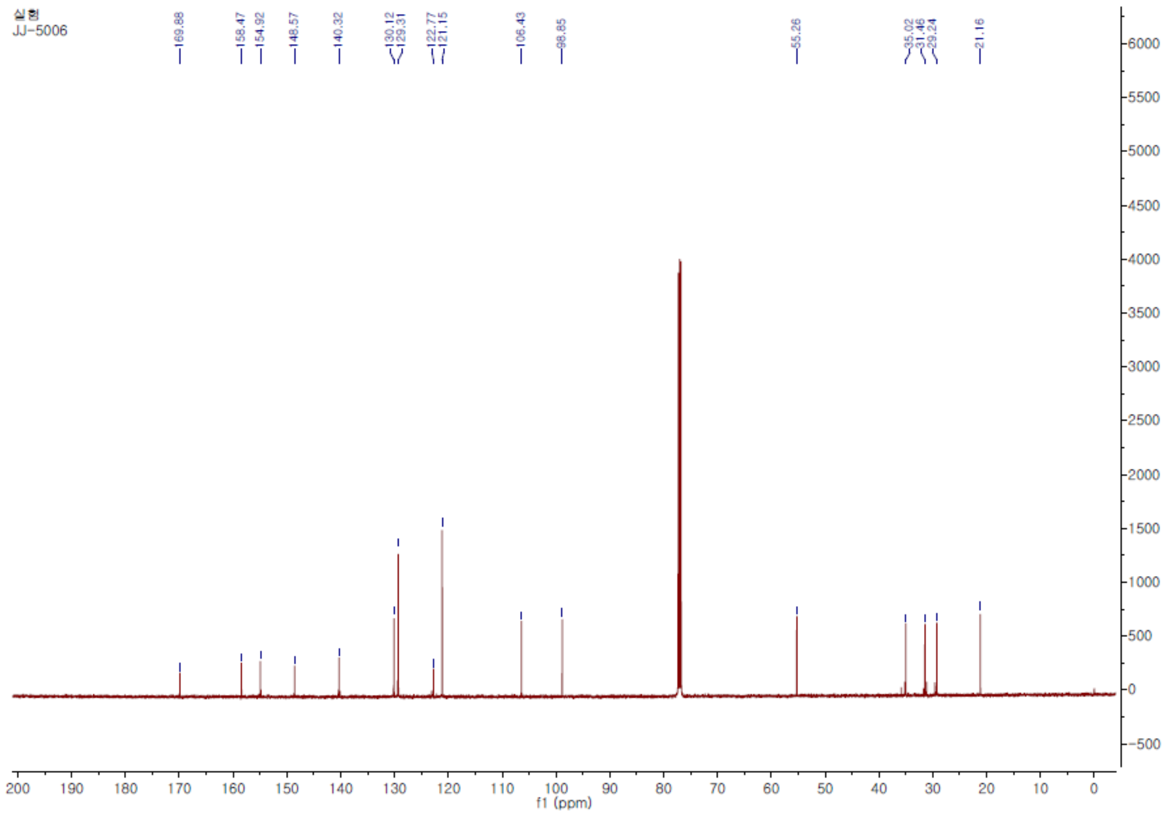

Figure S16. ^{13}C-NMR spectra of **10**.

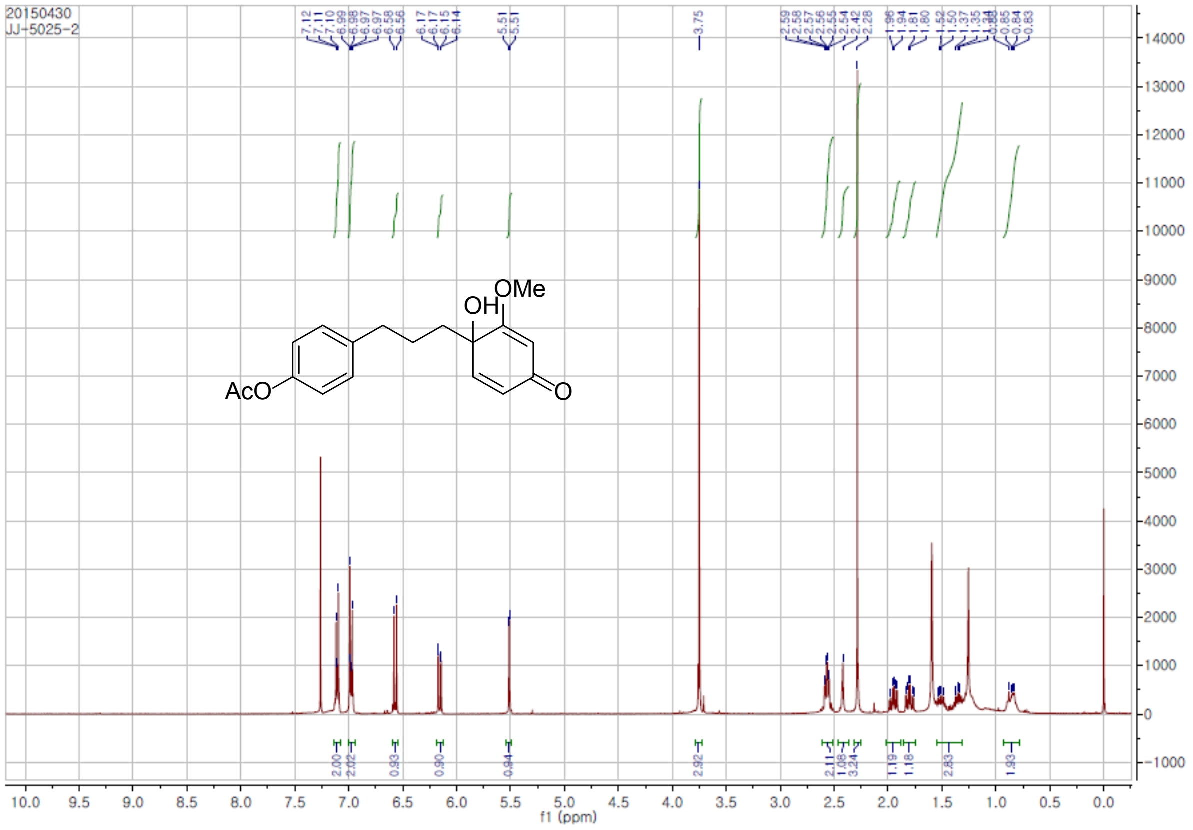

Figure S17. ^{1}H-NMR spectra of **11**.
